# Prenatal stress alters amygdala functional connectivity in preterm neonates

**DOI:** 10.1016/j.nicl.2016.08.010

**Published:** 2016-08-10

**Authors:** Dustin Scheinost, Soo Hyun Kwon, Cheryl Lacadie, Gordon Sze, Rajita Sinha, R. Todd Constable, Laura R. Ment

**Affiliations:** a Department of Radiology and Biomedical Imaging, Yale School of Medicine, New Haven, CT, United States; b Department of Pediatrics, Yale School of Medicine, New Haven, CT, United States; c Department of Psychiatry, Yale School of Medicine, New Haven, CT, United States; d Department of Child Study, Yale School of Medicine, New Haven, CT, United States; e Department of Neuroscience, Yale School of Medicine, New Haven, CT, United States; f Department of Neurosurgery, Yale School of Medicine, New Haven, CT, United States; g Department of Neurology, Yale School of Medicine, New Haven, CT, United States

**Keywords:** Amygdala, Prenatal stress, Very preterm, Infant brain, Functional connectivity

## Abstract

Exposure to prenatal and early-life stress results in alterations in neural connectivity and an increased risk for neuropsychiatric disorders. In particular, alterations in amygdala connectivity have emerged as a common effect across several recent studies. However, the impact of prenatal stress exposure on the functional organization of the amygdala has yet to be explored in the prematurely-born, a population at high risk for neuropsychiatric disorders.

We test the hypothesis that preterm birth and prenatal exposure to maternal stress alter functional connectivity of the amygdala using two independent cohorts. The first cohort is used to establish the effects of preterm birth and consists of 12 very preterm neonates and 25 term controls, all without prenatal stress exposure. The second is analyzed to establish the effects of prenatal stress exposure and consists of 16 extremely preterm neonates with prenatal stress exposure and 10 extremely preterm neonates with no known prenatal stress exposure. Standard resting-state functional magnetic resonance imaging and seed connectivity methods are used.

When compared to term controls, very preterm neonates show significantly reduced connectivity between the amygdala and the thalamus, the hypothalamus, the brainstem, and the insula (p < 0.05). Similarly, when compared to extremely preterm neonates without exposure to prenatal stress, extremely preterm neonates with exposure to prenatal stress show significantly less connectivity between the left amygdala and the thalamus, the hypothalamus, and the peristriate cortex (p < 0.05). Exploratory analysis of the combined cohorts suggests additive effects of prenatal stress on alterations in amygdala connectivity associated with preterm birth.

Functional connectivity from the amygdala to other subcortical regions is decreased in preterm neonates compared to term controls. In addition, these data, for the first time, suggest that prenatal stress exposure amplifies these decreases.

## Highlights

• Investigate effects of prenatal stress in extremely preterm neonates using rs-fMRI
• Amygdala connectivity is altered by both preterm birth and prenatal stress.
• Amygdala-subcortical connectivity the regions is decreased in very preterm neonates.
• Prenatal stress additionally reduces this connectivity in extremely preterm neonates.

## 1 Introduction

Preterm birth, birth before 37 weeks gestation, alters connectivity in the developing brain (Allievi et al., 2016, Karolis et al., 2016, Kwon et al., 2016, Smyser et al., 2016). While these alterations have been largely attributed to postnatal perturbations (Brummelte et al., 2012, Pineda et al., 2014, Rogers et al., 2016, Smith et al., 2011, Smyser et al., 2013), emerging data suggest that prenatal exposure to maternal stress may also play a role (Bronson and Bale, 2014, Chen and Baram, 2016, Constantinof et al., 2015, Provencal and Binder, 2015). Stress is a signal in response to challenging and uncontrollable adverse events and perceived threat (McEwen, 2002, Sinha, 2008). For typically developing term neonates, exposure to early life stress is a risk factor for neuropsychiatric disorders ranging from autism spectrum disorder and ADHD to depression and schizophrenia (Brown, 2012, Chin-Lun Hung et al., 2015, Entringer et al., 2015, Fox et al., 2010, Howerton and Bale, 2012, Khashan et al., 2008, Kinney et al., 2008, Koenig et al., 2002, Li et al., 2010, Pearson et al., 2013, Shonkoff et al., 2012, Talge et al., 2007). In contrast, the impact of prenatal stress on the connectome of the prematurely-born remains largely unexplored.

Stress-related symptoms of anxiety and depression are common in pregnant women (Burns et al., 2015), and epidemiologic studies suggest that not only is prenatal stress associated with preterm birth and low birth weight, it may also alter brain development (Baron et al., 2015, Burns et al., 2015, Castro et al., 2015, Robbins et al., 2014). In a broad range of preclinical and clinical studies, prenatal exposure to stress-related conditions has been shown to potently stimulate biological stress pathways,(Constantinof et al., 2015) alter synaptogenesis (Bale et al., 2010, Singh-Taylor et al., 2015) and result in changes in structural brain development (Qiu et al., 2015, Qiu et al., 2013, Rifkin-Graboi et al., 2013, Rifkin-Graboi et al., 2015, Van Dam et al., 2014).

Most MRI studies demonstrating the effect of stress during gestation and early life have been conducted in older subjects with alterations of amygdala structure and function being a common effect across studies (Buss et al., 2012, Gee et al., 2013a, Malter Cohen et al., 2013). Similarly, emerging reports of infants evaluated during the first postnatal year also highlight the vulnerability of the amygdala to early life stress. At 1–2 weeks of age, healthy term neonates with prenatal maternal depression exposure show lower fractional anisotropy, a measure of microstructural maturation, in the right amygdala compared to infants with no exposure (Rifkin-Graboi et al., 2013). Six month old infants born to mothers with high prenatal depressive symptoms show greater functional connectivity of the left amygdala with the left temporal cortex and insula, as well as the bilateral anterior cingulate, medial orbitofrontal and ventromedial prefrontal cortices, consistent with the pattern found in older children and adults with depressive disorders.(Qiu et al., 2015) Finally, infants between 6 and 12 months of age show increasing connectivity between the posterior cingulate and the amygdala with increasing inter-parental conflict and negative emotionality (Graham et al., 2015). However, amygdala functional connectivity has been less well-studied in those prematurely born. Preliminary findings in preterm neonates (Rogers et al., 2015) and young adults (Papini et al., 2014) suggest reduced amygdala connectivity to subcortical, limbic, and frontal regions.

Following these studies, using resting-state functional MRI (fMRI), we test the hypothesis that prenatal exposure to maternal stress reduces functional connectivity of the amygdala with these regions in preterm infants. First, we establish normal amygdala functional networks in neonates. Next, we show that at term equivalent age (TEA; 40–44 weeks postmenstrual age) very preterm neonates exhibit reduced amygdala connectivity to subcortical regions. Then, in an independent cohort of extremely preterm neonates imaged at 36–43 weeks postmenstrual age, we show that preterm neonates exposed to prenatal stress exhibit reduced amygdala connectivity to similar subcortical regions. Finally, as exploratory analysis, we combine the cohorts to show a synergistic relationship between prenatal stress and preterm birth, resulting in greatest alterations of amygdala connectivity for preterm neonates with prenatal stress exposure. Together, these results suggest that reduction in amygdala connectivity associated with preterm birth is moderated by exposure to prenatal stress.

## 2 Methods

This study was approved by the Yale University Human Investigation Committee.

### 2.1 Participants

#### 2.1.1 Cohort 1

Very preterm neonates born (< 32 weeks gestation) with a birth weight (BW) between 500 and 1500 g and healthy term controls born between 37 and 41 weeks postmenstrual age (PMA) were eligible for the protocol and prospectively enrolled between 9-01-10 and 10-31-13. All were inborn and appropriate for gestational age (AGA). Exclusion criteria included evidence of congenital infections, congenital malformations and/or chromosomal disorders, seizures, intraventricular hemorrhage (IVH), periventricular leukomalacia or focal abnormalities on a previous or study MRI. In addition, all preterm participants had normal cranial ultrasounds prior to 7–14 days of life. This cohort has been previously published (Kwon et al., 2014, Scheinost et al., 2016) and includes 25 term controls and 12 preterm neonates. Very preterm neonates were scanned at term equivalent age. No neonate in this cohort had documented exposure to prenatal stress.

#### 2.1.2 Cohort 2

Extremely preterm neonates (< 28 weeks gestation) were eligible for the protocol and prospectively enrolled between 4-01-12 and 10-31-15. All were inborn and appropriate for gestational age. Psychological stress during pregnancy was obtained by retrospective electronic chart review. For the purposes of this preliminary report, we employed a binary classification of prenatal stress exposure as documented by the attending physician's diagnosis of depression and/or anxiety in the maternal medical chart. Exclusion criteria included maternal pharmacologic treatment for these conditions as well as neonatal evidence of congenital infections, congenital malformations, chromosomal disorders, seizures, or major abnormalities on MRI, including intraventricular hemorrhage, periventricular leukomalacia, and ventriculomegaly. In addition, all preterm participants had normal cranial ultrasounds within the first 7–14 days of life. Twenty eight extremely preterm neonates met all inclusion criteria, and data from 10 extremely preterm neonates with prenatal stress exposure and 16 extremely preterm neonates with no stress exposure were analyzed after exclusion of 2 neonates due to motion artifact. All connectivity processing was performed blinded to prenatal stress exposure for Cohort 2.

### 2.2 Imaging parameters

Subjects were scanned without sedation using a feed-and-wrap protocol in either a 3T Siemens (Erlangen, Germany) TIM Trio MR system for Cohort 1 or a 3T Siemens Verio Clinical system for Cohort 2. Both cohorts were scanned using a 32-channel parallel receiver head coil. Localizer images were acquired for prescribing the functional image volumes, aligning the seventh or eighth slice parallel to the plane transecting the anterior and posterior commissures. T1-weighted 2D anatomical images were collected (TR = 300 ms, TE = 2.47 ms, FoV = 220 mm, matrix size = 256 × 256, slice thickness = 4 mm, Flip Angle = 60°, Bandwidth = 300 Hz/pixel with 25 slices) with 25 AC-PC aligned axial-oblique slices in addition to 3D anatomical scans using Magnetization Prepared Rapid Gradient Echo (MPRAGE) (176 contiguous sagittal slices, slice thickness = 1 mm, matrix size = 256 × 256, FoV = 256 mm, TR = 2530 ms, TE = 2.77, Flip Angle = 7°, Bandwidth = 179 Hz/pixel). Similarly for Cohort 2, in-plane resolution and the number of slices were modified in response to time limitations. After these structural images, acquisition of functional data began in the same slice locations as the axial-oblique T1-weighted data. Similarly for Cohort 2, in-plane resolution and number of slices were modified based on time limitations of the scanner. Functional images were collected using an echo-planar image gradient echo pulse sequence (TR = 1500 ms, TE = 27 ms, FoV = 220 mm, matrix size = 64 × 64, slice thickness = 4 mm, Flip Angle = 60°, Bandwidth = 2520 Hz/pixel, 25 slices). Two to three functional runs were collected for Cohort 1 and one functional run was collected for Cohort 2. Functional runs consisted of 186 volumes (approximately 5 min scan length) for Cohort 1 and 235 volumes for Cohort 2 after the first 6 volumes were removed to allow the magnetization to reach the steady-state.

### 2.3 Connectivity preprocessing

Images were slice-time and motion corrected using SPM5 (http://www.fil.ion.ucl.ac.uk/spm/software/spm5/). All further analyses were performed using BioImage Suite.(Joshi et al., 2011). Several covariates of no interest were regressed from the data including linear and quadratic drift, six rigid-body motion parameters, mean cerebral-spinal-fluid (CSF) signal, mean white-matter signal, and overall global signal. The data were temporally smoothed with a zero mean unit variance Gaussian filter (approximate cutoff frequency = 0.12 Hz). A gray matter mask was applied to the data so only voxels in the gray matter were used in the calculation. The gray matter mask was a standard gray matter mask in MNI space warped to our neonatal template brain (see below for details on the template brain). As the gray matter is larger in the adult MNI template, this ensures full coverage of the gray matter even in the presence of registration and segmentation errors. Spurious signals that may be incorrectly labeled as gray matter are removed by the nuisance regression of the CSF, white matter, and global signal. Blocks of data with a displacement > 1.5 mm or a rotation > 2° of rotation were removed. All subjects had at least 2.5 min of resting state data. Finally, as group differences in motion have been shown to confound connectivity studies (Van Dijk et al., 2012), we calculated the average frame-to-frame displacement for each participant's data. There were no significant differences for motion when term infants were compared to very preterm neonates (term: 0.05 ± 0.01, preterm: 0.05 ± 0.03, p = 0.80) or when extremely preterm neonates with prenatal stress exposure were compared to those with no prenatal stress exposure (stress: 0.07 ± 0.06, no stress: 0.06 ± 0.03, p = 0.68).

### 2.4 Amygdala seed connectivity

**Fig. 1:**
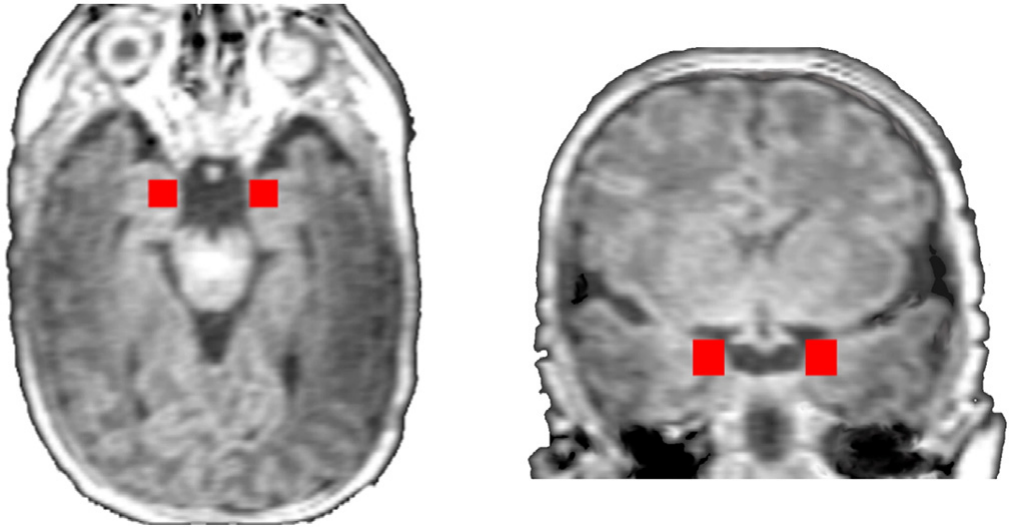
Spatial location of the right and left amygdala seeds. The right and left amygdala seeds are shown on an axial and a coronal slice in red. The seed was defined as 10 mm cube in common space. (For interpretation of the references to color in this figure legend, the reader is referred to the web version of this article.)

Connectivity analyses were performed to determine whole brain seed connectivity from the left and right amygdala (shown in Fig. 1). The amygdala seeds were defined on the reference brain and transformed back (via the inverse of the transforms described below) into individual participant space. The time course of the reference region in a given participant was then computed as the average time course across all voxels in the reference region. This time course was correlated with the time course for every other voxel in the gray matter to create a map of r-values, reflecting seed-to-whole-brain connectivity. These r-values were transformed to z-values using Fisher's transform yielding one map for each seed and for each participant representing the strength of correlation to the amygdala seed.

### 2.5 Common space registration

To facilitate comparisons of imaging data, all single participant amygdala connectivity results were first spatially smoothed with a 6 mm Gaussian filter and warped to a common template space through the concatenation of a series of linear and non-linear registrations. The functional series were linearly registered to the T1 axial-oblique (2D anatomical) image. The 2D anatomical image was linearly registered to the MPRAGE (3D anatomical) image. The 3D anatomical image was non-linearly registered to the template brain. For this study, the template brain was chosen from an independent healthy term neonate collected on the same scanner as Cohort 1. We selected a single representative infant brain as the template, rather than the brain generated by averaging all of the neonates brains, because a single brain has well-defined tissue interfaces that reduce errors during registration (Spann et al., 2014, Spann et al., 2015).

All transformation pairs were calculated independently and combined into a single transform warping the single participant results into common space. This single transformation allows the single participant images to be transformed to common space with only one transformation, reducing interpolation error. All transformations were estimated using the intensity-only component of the method implemented by BioImage Suite.

### 2.6 Statistical analyses

Demographic data were analyzed either using standard chi-squared test statistics or using Fisher's exact test for categorical data. Continuous-valued data were analyzed either using t-tests or using Mann-Whitney u-tests when a normal distribution could not be assumed to compare groups. All analyses were performed using SAS version 9.4 (Cary, NC). P-value of < 0.05 was considered to be statistically significant.

Imaging data were analyzed using voxels *t*-tests. Significance was assessed at a p < 0.05 for between group comparisons. All maps were corrected for multiple comparisons across gray matter using cluster-level correction estimated via Monte Carlo simulations. AFNI's 3dClustSim was used to estimate a cluster size of 4768.2 mm^3^ using 10,000 iterations, an initial p-value threshold of 0.05, the gray matter mask using in preprocessing, and smoothness values estimated from the residuals using 3dFWHMx.

## 3 Results

### 3.1 Subject characteristics

Participant characteristics for Cohort 1 are presented in Table 1. There were no significant differences in sex, race, and postmenstrual age at scan. Characteristics for the participants in Cohort 2 are shown in Table 2. Preterm neonates with no prenatal stress exposure had significantly lower gestational ages and birth weights compared to the group with prenatal stress exposure (p = 0.04 and p = 0.01, respectively), but there were no significant differences in sex, race, postmenstrual age at scan and perinatal morbidities. When combined across cohorts, for the preterm neonates, there were no differences in postmenstrual age at birth, birth weight, or sex distribution (p = 0.30, p = 0.17, and p = 0.16, respectively). However, there was a significant difference in postmenstrual age at scan (p = 0.003). For exploratory analysis combed across cohorts, scanner and postmenstrual age at scan were included as covariates.

### 3.2 Neonatal amygdala connectivity

**Fig. 2:**
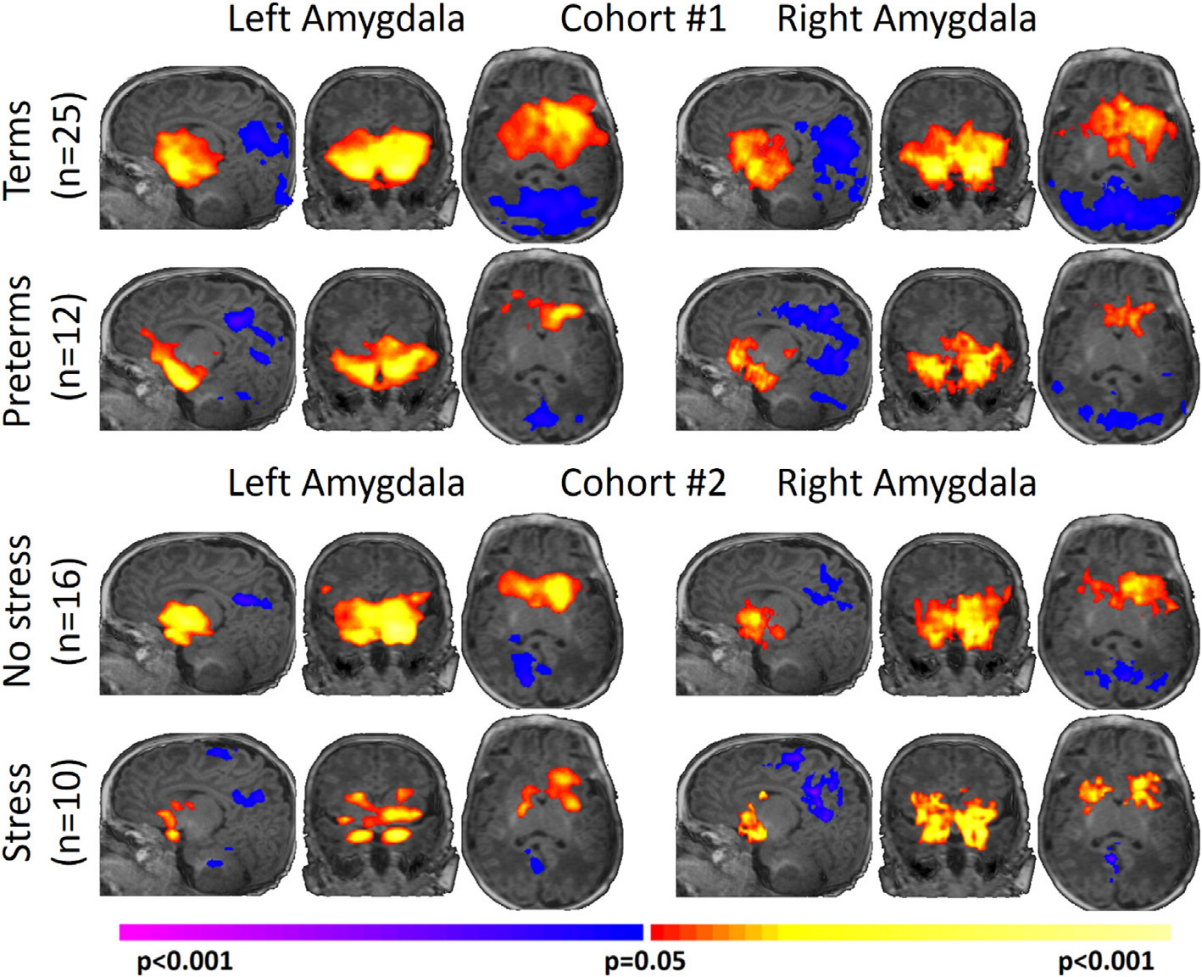
Neonatal amygdala connectivity for each study group. All four study groups displayed similar patterns, albeit at different strengths, of left and right amygdala connectivity with positive connectivity to subcortical regions and negative connectivity to cortical regions. Results are shown at p < 0.05, corrected. Warm colors represent areas of positive connectivity to the amygdala. Cool colors represent areas of negative connectivity to the amygdala. (For interpretation of the references to color in this figure legend, the reader is referred to the web version of this article.)

As shown in Fig. 2, all four study groups displayed similar patterns, albeit at different strengths, of left and right amygdala connectivity (p < 0.05, corrected). Overall, the left and right amygdala showed significant positive connectivity to subcortical regions including the contralateral amygdala, the hippocampus, the brainstem, the insula, the hypothalamus, and the thalamus. In addition, the left and right amygdala showed significant negative connectivity to the posterior cingulate cortex, precuneus, and the visual cortex.

### 3.3 Cohort 1: very preterm neonates versus term controls

**Fig. 3:**
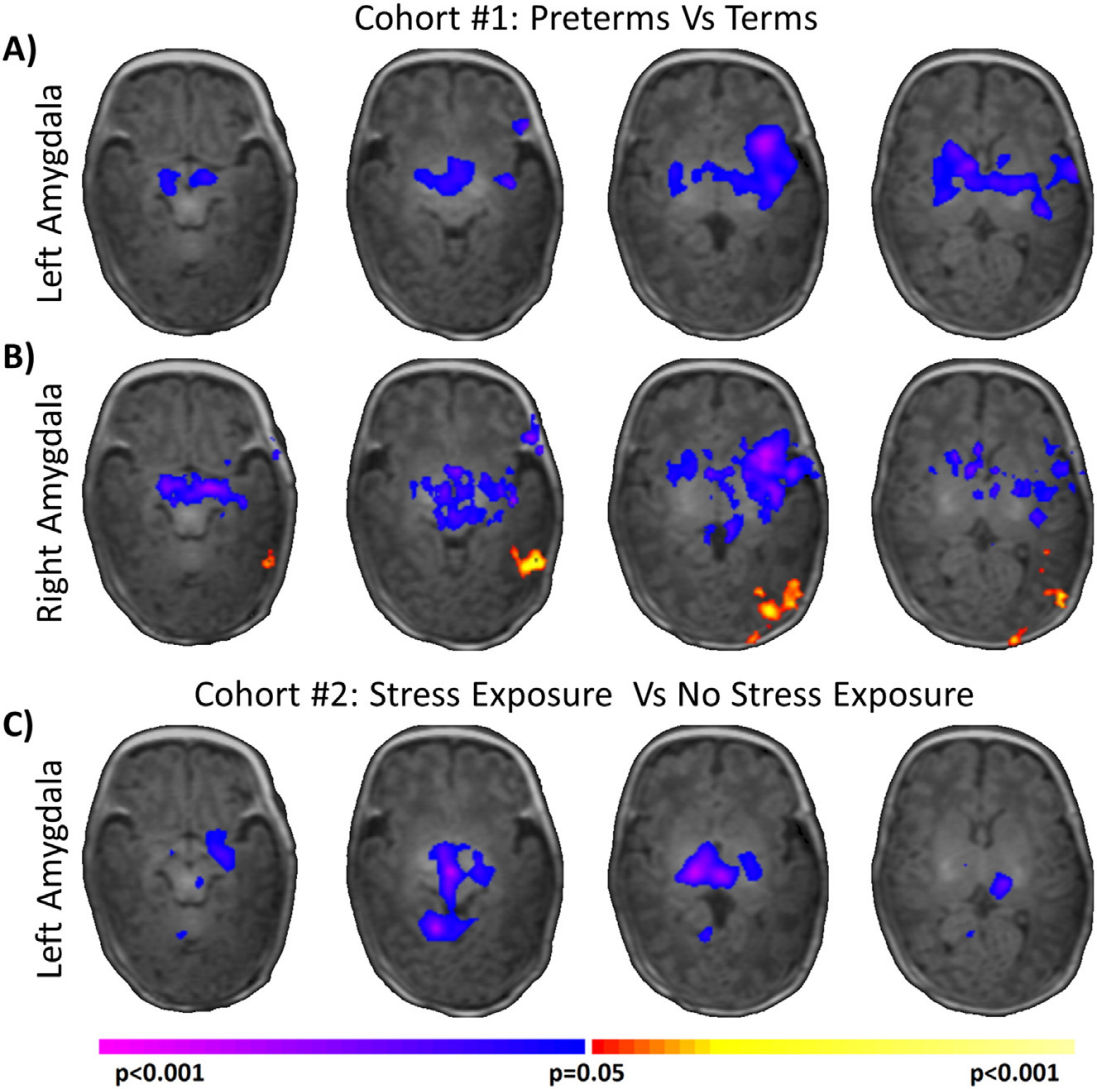
Comparison of amygdala connectivity between study groups. Comparisons of very preterm neonates to term controls suggest widespread, significant (p < 0.05, corrected) alteration of the neonatal amygdala network for both the A) left amygdala and B) right amygdala. C) Additionally, comparisons of extremely preterm neonates with and without prenatal stress exposure suggested prenatal stress significantly (p < 0.05, corrected) further reduces the strength of the amygdala network. Warm colors represent areas of greater connectivity for A–B) the preterm group or C) the stress group. Cool colors represent areas of reduced connectivity for A–B) the preterm group or C) the stress group. (For interpretation of the references to color in this figure legend, the reader is referred to the web version of this article.)

As shown in Figs. 3A and B, when compared to term controls, very preterm neonates exhibited widespread alterations in neonatal amygdala networks for both the left and right amygdala. For both seeds, significantly (p < 0.05, corrected) reduced amygdala connectivity with the brainstem, insula, hypothalamus and thalamus was observed in very preterm neonates. In addition, for the right amygdala seed, very preterm neonates exhibited significantly (p < 0.05, corrected) increased (less negative) connectivity to the occipital lobe when compared to term controls. A similar but not significant cluster was also observed using the left amygdala seed. As sex was significantly different between the two study groups, we repeated our analysis controlling for sex and found no changes in the results. Additionally, no significant main effects of sex or sex-by-group interaction were found.

### 3.4 Cohort 2: Prenatal stress exposure versus no prenatal stress exposure

**Fig. 4:**
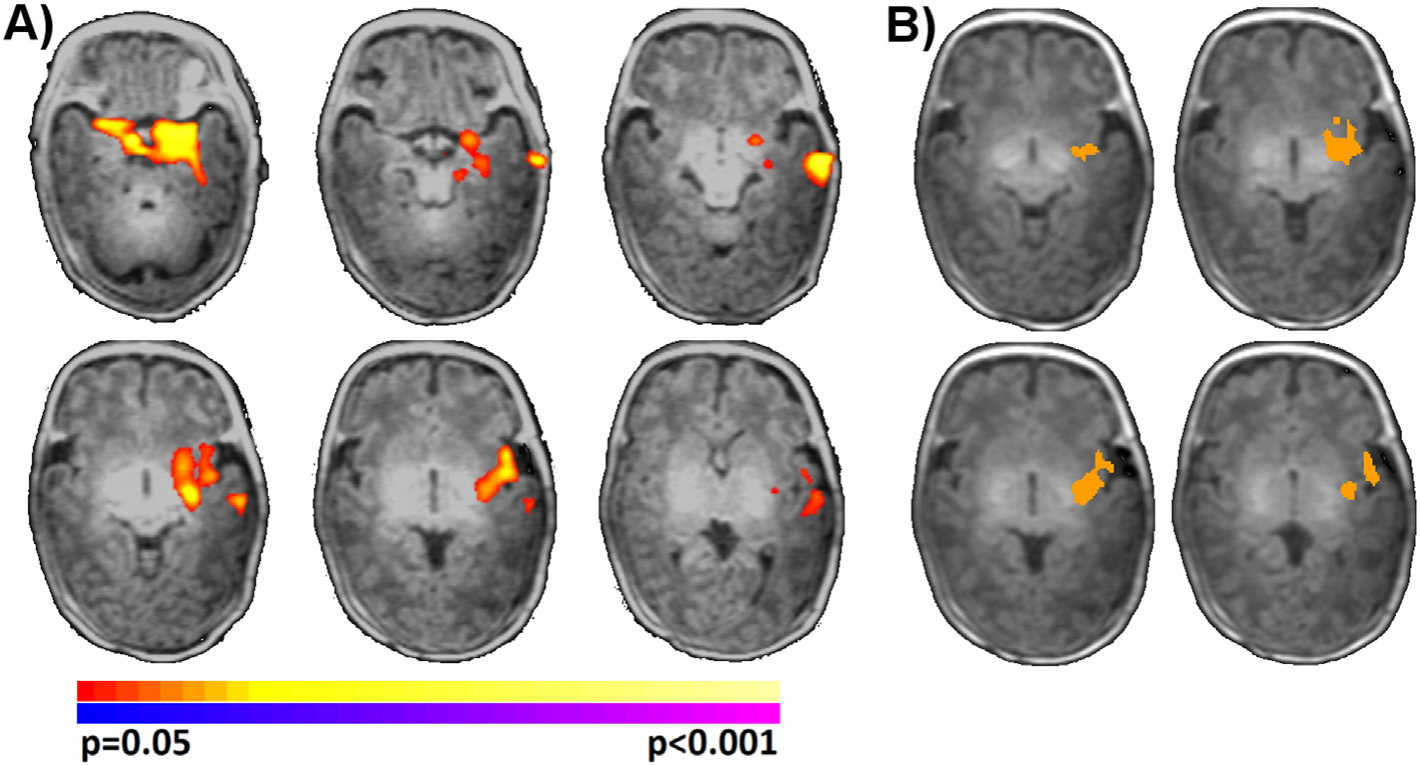
Main effect of gestation age at birth for amygdala connectivity. A) Significant (p < 0.05, corrected) main effect of GA at birth was observed regions independent of main effect of prenatal stress exposure for Cohort 2. Warm colors represent areas of increasing connectivity with increasing GA at birth. B) The direction of this effect was consistent and overlapped with the main effects of group from Cohort 1, where reduced GA at birth (the preterm group) was associated with reduced connectivity to the same regions. Overlap of Fig. 3A and 4A shown in orange. (For interpretation of the references to color in this figure legend, the reader is referred to the web version of this article.)

As shown in Fig. 3C, extremely preterm neonates with prenatal stress exposure exhibited significantly (p < 0.05, corrected) reduced left amygdala connectivity to the brainstem, the fusiform, the hypothalamus, and the thalamus when compared to extremely preterm neonates without prenatal stress exposure. When using the right amygdala seed, a similar subcortical cluster that did not reach significance was observed. As there was a significant difference in GA at birth and birth weight for the two study groups in this cohort, we repeated our main analysis independently covarying for GA at birth and birth weight and found no changes in the results. In addition, a main effect of GA at birth was observed in the left temporal lobe and left insula, such that greater GA at birth was associated with greater left amygdala connectivity to these regions (Fig. 4A). The direction of this effect was consistent and overlapped with the main effect of group from Cohort 1, where reduced GA at birth (the preterm group) was associated with reduced connectivity to the same regions (Fig. 4B). Taken together, these results suggest that a portion of the neonatal amygdala network (connections to temporal lobe and insula) are modulated by GA at birth and that a different portion of the neonatal amygdala network is modulated by other factors including prenatal stress exposure.

### 3.5 Exploratory connectivity combined across cohorts

**Fig. 5:**
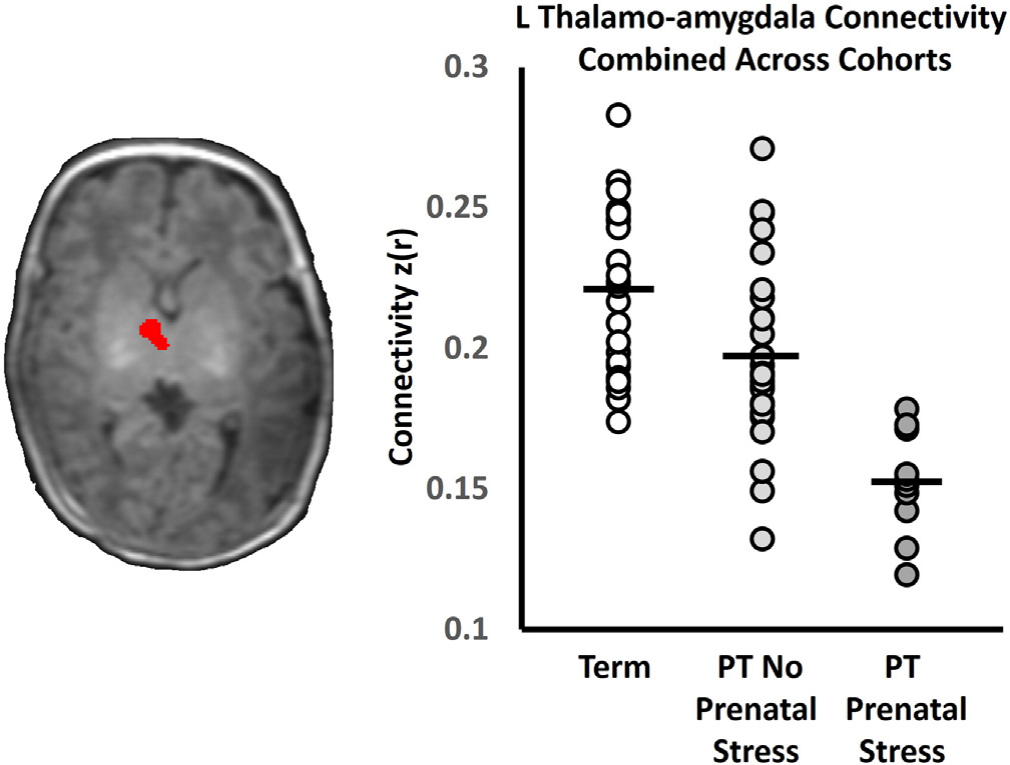
Left Thalamo-amygdala connectivity combined across cohorts. Altered thalalmo-amygdala connectivity was common across both cohorts and a conjunction analysis was used to define a region of interest for further analysis (shown in red on the axial slice). When data from both cohorts was combined, left thalamo-amygdala connectivity showed the hypothesized reduction due to preterm birth and preterm birth with prenatal stress exposure. Term control data are shown as white circles. Data from the preterm neonates without prenatal stress exposure are shown as light gray circles. Data from the preterm neonates with prenatal stress exposure are shown as dark gray circles. Black bars represent the group means. (For interpretation of the references to color in this figure legend, the reader is referred to the web version of this article.)

When taken together, the independent results from each cohort suggest that preterm birth is associated with reduced strength of the neonatal amygdala network and that prenatal stress further reduces the network's strength. Combining cohorts, we tested the hypothesis that term controls would display the greatest amygdala connectivity, followed by preterm neonates without stress exposure and, finally, by preterm neonates with stress exposure. We extracted left thalamo-amygdala connectivity from a thalamic region of interest, defined from the conjunction of the results shown in Fig. 3A and C. To account for differences in scanner, postmenstrual age at scan. And sex, a linear model with these factors was fit to the data, and corrected thalamo-amygdala connectivity values were calculated from the residuals. As shown in Fig. 5, the thalamo-amygdala connectivity demonstrated the hypothesized relationship with term controls exhibiting the greatest connectivity and preterm neonates with prenatal stress exposure exhibiting the weakest connectivity. All pairwise comparisons between each group's thalamo-amygdala connectivity were significantly different (p < 0.05).

## 4 Discussion

Amygdala circuitry is critical in developmental psychopathology and appears to be vulnerable to early life stress. Using two independent cohorts, we provide evidence that preterm birth and prenatal exposure to maternal stress alter amygdala connectivity assessed in the neonatal period. In the first cohort, we demonstrate that very preterm neonates at TEA have reduced amygdala connectivity to subcortical structures when compared to term controls. In the second, we show that extremely preterm neonates exposed to prenatal maternal stress have reduced amygdala connectivity to similar subcortical structures when compared to extremely preterm neonates without prenatal stress exposure. When combined, exploratory analysis suggests that prenatal stress further reduces amygdala connectivity in preterm neonates. These results highlight the importance of prenatal stress and amygdala connectivity in preterm birth, a population at high risk for neuropsychiatric disorders.

While alterations in neural connectivity are the hallmark of the prematurely-born (Kwon et al., 2016), these changes have been largely attributed to the numerous neonatal sequelae common to the infants born preterm. Our results add to the emerging data suggesting that prenatal exposure to maternal stress may also play a role in preterm birth outcomes.(Bronson and Bale, 2014, Chen and Baram, 2016, Constantinof et al., 2015, Provencal and Binder, 2015). Preclinical models suggest that prenatal stress results in early disruption of GABAergic progenitor migration (Stevens et al., 2013) and impairs synaptogenesis (Bale et al., 2010, Howerton and Bale, 2012). In addition, preclinical models suggest that amygdala connectivity is mediated by GABA transmission for stress-induced behavior (Andolina et al., 2013) and that prolonged stress enhances synaptic connectivity in the amygdala (Vyas et al., 2006). These processes are also known to be disrupted during preterm birth (Lubsen et al., 2011). Taken together, disruption of common processes may explain the similarity of results from the comparisons of the two cohorts and the possible synergistic effects of prenatal stress and preterm birth on amygdala connectivity.

Amygdala hypo-connectivity is characteristic of childhood neuropsychiatric diagnoses including autism spectrum and bipolar disorder (Ecker and Murphy, 2014, Pelphrey and Carter, 2008, Rich et al., 2008, von dem Hagen et al., 2013). Preterm neonates are reported to be at high risk for autism spectrum disorder (Fevang et al., 2016, Limperopoulos et al., 2008, Pritchard et al., 2016), with brain alterations in socio-emotional networks during neonatal period.(Padilla et al., 2015). Likewise, preterm adolescents have increased risk of socialization problems, a key component of autism spectrum disorders. These difficulties are associated with reduced structural covariance in these networks including the amygdala (Healy et al., 2013). A proposed model suggests that preterm birth leads to brain alterations in socio-emotional networks and links these alterations to future psychopathology (Montagna and Nosarti, 2016). Our results extend this model to include gestational exposures as factors that alter connectivity in socio-emotional networks. Further study of this circuit in preterm neonates may help elucidate the role of the amygdala in the development of future child and adult psychiatric disorders.

Across all study groups, the amygdala showed similar patterns of connectivity, albeit at different strengths. The amygdala was positively connected to subcortical regions including the hypothalamus, thalamus, insula, and brainstem and was negatively connected to cortical regions including precuneus, cuneus, and occipital lobe. These connectivity patterns are consistent with a previous reports of amygdala connectivity in neonates of a similar age (Rogers et al., 2015, Salzwedel et al., 2015) and at 6 months (Qiu et al., 2015). Notably, we did not observe connectivity between the amygdala and the medial prefrontal cortex/anterior cingulate, a connection that appears to be highly sensitive to early life adversities (Gee et al., 2013a, Malter Cohen et al., 2013, Qiu et al., 2015, Salzwedel et al., 2015, Thomason et al., 2015). The development of amygdala-mPFC connectivity is characterized by a reorganization from positive to negative connectivity across childhood through adolescence (Gee et al., 2013b). However, the development of amygdala-mPFC connectivity during the neonatal and early infancy period is less well characterized with negative (Rogers et al., 2015, Salzwedel et al., 2015), positive (Qiu et al., 2015), and no connectivity reported. Continued research is needed to map the developmental trajectories of amygdala-mPFC connectivity from the 3rd trimester to childhood.

The strengths of this study are the prospective data collection and well-defined subject population with no MRI evidence of brain injury. However, several weaknesses exist. First, we defined prenatal stress as a diagnosis of depression and/or anxiety from clinical chart review. It is difficult to fully disentangle the effects of prenatal maternal stress versus maternal depression and anxiety. In addition, other types of prenatal stress are unaccounted for in this analysis and some subjects may have been exposed to prenatal stress undocumented in the clinical charts. Second, our study consists of two cohorts, each able to capture only one effect of interest. Differences in PMA at scan and scanner effects between the cohorts may be non-linear and difficult to model, especially in a whole brain analysis. For example, different scanners may produce images with inherently different smoothness (Friedman et al., 2006) and differences in smoothness can confound connectivity analyses (Scheinost et al., 2014). For these reasons, we present analysis combining the cohorts as exploratory. Additionally, the link between preterm birth, prenatal stress exposure, and offspring outcome may be moderated by fetal sex (Ingemarsson, 2003, Wainstock et al., 2015). However, we were not able to detect sex differences in our cohort, which may be due to a significant difference in the number of male and female very preterm neonates in Cohort 1. Other weaknesses include the as yet unavailable behavioral testing data and longitudinal imaging studies assessing the impact of prenatal stress exposure on neural networks in the fetal developing brain.

There is increasing recognition that changes in women's stress-based physiology may influence fetal neural development, (Doyle et al., 2015) and the data we report extends previously published work to a vulnerable population, extremely preterm neonates. Those exposed to prenatal stress show reduced connectivity of amygdala functional networks. Future work should not only define the behavioral phenotype of affected infants through grade school and beyond, but also inform specific targets and timing of future neuroprotective strategies.

## Figures and Tables

**Table 1:** Characteristics of study participants in Cohort 1.

	Preterm (n = 12)	Term (n = 25)	p-Value
Postmenstrual age at birth (weeks)	27.4 ± 2.2	40.0 ± 0.9	< 0.001
Birth weight (grams)	1015 ± 330	3350 ± 380	< 0.001
Postmenstrual age at scan (weeks)	42.3 ± 0.9	42.3 ± 1.3	0.96
Male sex	3 (25%)	15 (60%)	0.08
Non-white	6 (50%)	7 (28%)	0.27
Bronchopulmonary dysplasia	3 (25%)		
Retinopathy of prematurity	4 (33%)		
Late-onset sepsis	2 (17%)		

Values are mean ± SD.

**Table 2:** Characteristics of study participants in Cohort 2.

	Prenatal stress exposure (n = 10)	No prenatal stress exposure (n = 16)	p-Value
Postmenstrual age at birth (weeks)	27.2 ± 0.4	26.4 ± 1.1	0.04
Birth weight (grams)	1020 ± 110	830 ± 200	0.01
Postmenstrual age at scan (weeks)	37.9 ± 1.7	37.5 ± 2.5	0.67
Male sex	5 (50%)	9 (56%)	0.70
Non-white	1 (17%)	5 (56%)	0.35
Bronchopulmonary dysplasia	5 (50%)	7 (44%)	1
Retinopathy of prematurity	4 (40%)	7 (44%)	1
Late-onset sepsis	0 (0%)	0 (0%)	1

Values are mean ± SD.
